# Simultaneous Cranioplasty and Ventriculoperitoneal Shunt Placement After Decompressive Craniectomy in Children: A Single-Center Experience

**DOI:** 10.3390/jcm15176777

**Published:** 2026-08-31

**Authors:** Duygu Dolen Burak, Mustafa Selim Şahin, Cafer Ikbal Gülsever, Duran Sahin, Tugrul Cem Unal, Ilyas Dolas, Pulat Akin Sabanci, Yavuz Aras, Aydın Aydoseli, Altay Sencer

**Affiliations:** 1 Department of Neurosurgery, Istanbul Faculty of Medicine, Istanbul University, 34093 Istanbul, Türkiye; 2 Department of Neurosurgery, Hakkari State Hospital, Ministry of Health, 30000 Hakkari, Türkiye; 3 Department of Neurosurgery, Medical Faculty, Istanbul Bilim University, 34394 Istanbul, Türkiye

**Keywords:** cranioplasty, decompressive craniectomy, hydrocephalus, pediatric neurosurgery, ventriculoperitoneal shunt, simultaneous surgery, staged surgery

## Abstract

**Background:** The optimal sequencing of cranioplasty (CP) and ventriculoperitoneal shunt (VPS) placement after decompressive craniectomy (DC) remains uncertain, particularly in pediatric patients. This study aimed to describe our pediatric experience and provide an exploratory comparison of simultaneous and staged procedures. **Methods:** We retrospectively reviewed the medical records of 12 pediatric patients who developed hydrocephalus after DC at a single tertiary center between 2015 and 2023. Six patients underwent simultaneous CP and VPS placement, while six underwent staged VPS placement followed by CP. Demographic and clinical characteristics, surgical timing, postoperative complications, and neurological outcomes were compared. Given the small sample size, analyses were exploratory and primarily descriptive. **Results:** Postoperative complications occurred in two patients in each group (33.3%), including one wound infection and one shunt dysfunction requiring revision. No intracranial hemorrhage or deep shunt infection occurred in either group. The complication rate did not differ between the groups (*p* = 1.000). The median final Glasgow Outcome Scale score was 2.5 (range, 2–3) in the simultaneous group and 3.0 (range, 1–5) in the staged group (*p* = 0.457). Neurological outcomes likely reflected the severity of the underlying brain injury; however, the small sample size precluded adjustment for prognostic factors. **Conclusion:** In this small retrospective pediatric cohort, simultaneous CP and VPS placement was technically feasible, but postoperative complications occurred in one-third of patients. No significant differences were observed between the simultaneous and staged groups; however, these findings should not be interpreted as evidence of equivalence because of the limited sample size and non-randomized design. Surgical strategy should be individualized, and larger multicenter pediatric studies are needed.

## 1. Introduction

Decompressive craniectomy (DC) is a lifesaving procedure for refractory intracranial hypertension but is frequently complicated by post-craniectomy hydrocephalus and the need for delayed cranial reconstruction. Altered cerebrospinal fluid (CSF) dynamics and impaired intracranial compliance contribute to hydrocephalus, which may negatively affect neurological recovery if left untreated [1].

Cranioplasty (CP) restores cranial protection and contour and may improve cerebral perfusion, intracranial compliance, and CSF circulation. Ventriculoperitoneal shunt (VPS) placement remains the standard treatment for persistent hydrocephalus. When both procedures are required, they may be performed either simultaneously or in separate stages. Simultaneous surgery may reduce cumulative anesthesia exposure, the number of hospital admissions, and delays in rehabilitation. Conversely, staged surgery may reduce the risks associated with combining cranial reconstruction and permanent CSF diversion in a single operation [2].

The optimal strategy remains controversial. Several studies have reported higher rates of infection, hemorrhage, subdural fluid collection, and overall complications following simultaneous surgery, whereas others have found no significant differences between the two approaches. Most available evidence is derived from adult populations. Its applicability to children remains uncertain because skull growth, age-dependent brain compliance, bone-flap behavior, and long-term shunt dependency may influence surgical outcomes [3].

This study aimed to provide an exploratory comparison of simultaneous and staged CP and VPS placement in a pediatric cohort and to evaluate the findings within the context of the available literature.

## 2. Materials and Methods

### 2.1. Study Design and Ethics

This retrospective single-center study reviewed the medical records of patients who underwent simultaneous cranioplasty (CP) and ventriculoperitoneal shunt (VPS) placement following decompressive craniectomy (DC) and staged surgery between January 2015 and December 2023. Hydrocephalus was diagnosed based on progressive ventriculomegaly on serial neuroimaging, defined by an Evans Index > 0.30, together with the treating neurosurgical team’s clinical judgment and the need for permanent cerebrospinal fluid (CSF) diversion. Institutional ethics committee approval was obtained (Approval No: 2025/1884), and the requirement for informed consent was waived due to the retrospective design.

### 2.2. Patient Selection

Patients who underwent simultaneous cranioplasty (CP) and ventriculoperitoneal shunt (VPS) placement following DC constituted the study group. To provide a comparative cohort, pediatric patients managed with staged VPS followed by cranioplasty during the same study period were retrospectively identified from the institutional database and included as the control group. Patients with incomplete clinical or radiological records, insufficient follow-up data, or previous CSF diversion procedures before decompressive craniectomy were excluded.

### 2.3. Selection of Surgical Strategy

No predefined institutional protocol was used to assign patients to simultaneous or staged surgery. The surgical strategy was determined individually by the treating neurosurgical team according to the patient’s clinical condition, radiological findings, infection risk, and suitability for cranial reconstruction. Simultaneous CP and VPS placement was considered in clinically stable patients with established shunt-dependent hydrocephalus, no evidence of active systemic or cranial infection, and adequate scalp and bone-flap conditions for reconstruction. A staged approach was preferred when the patient was clinically unstable, infection risk was considered elevated, the requirement for permanent CSF diversion remained uncertain, or the local wound, scalp, or bone-flap condition was unsuitable for immediate cranioplasty.

### 2.4. Surgical Technique

All cranioplasties were performed using the patients’ autologous bone flaps, which had been preserved according to the institutional protocol. Ventriculoperitoneal shunts were inserted through frontal or parietal burr holes using programmable Medtronic Strata^®^ valve systems(Medtronic, Minneapolis, MN, USA).

During simultaneous procedures, strict sterile precautions were observed, including complete separation of the cranial and abdominal operative fields. Standard perioperative antibiotic prophylaxis was administered in all patients. Initial programmable valve settings were selected at relatively high opening pressures to minimize early postoperative over-drainage and were subsequently adjusted according to the patients’ clinical and radiological course.

### 2.5. Statistical Analysis

Because of the pilot nature of the study and the limited sample size, statistical analyses were primarily descriptive. Continuous variables are presented as median (range) or mean ± standard deviation where appropriate, whereas categorical variables are presented as frequencies and percentages. Comparative analyses between the simultaneous and staged groups were performed to provide preliminary observations regarding clinical outcomes.

### 2.6. Data Collection

Demographic data, indication for DC, interval between DC and simultaneous surgery, postoperative complications, and length of hospital stay were recorded. Complications included surgical site infection, shunt malfunction or infection, intracranial hemorrhage, and subdural fluid collections requiring intervention. Neurological outcomes were assessed using the Glasgow Outcome Scale (GOS) at last follow-up.

### 2.7. Literature Review

A structured literature search was conducted using PubMed, Scopus, and Web of Science for studies published between January 2010 and December 2024. Search terms included “decompressive craniectomy,” “cranioplasty,” “ventriculoperitoneal shunt,” “hydrocephalus,” “simultaneous,” and “staged.” Comparative cohort studies, systematic reviews, and meta-analyses evaluating simultaneous versus staged procedures were included (Table 1).

## 3. Results

### 3.1. Patient Characteristics

A total of 12 pediatric patients who developed hydrocephalus following decompressive craniectomy (DC) were included in this pilot comparative cohort. Six patients underwent simultaneous cranioplasty (CP) and ventriculoperitoneal shunt (VPS) placement (simultaneous group), whereas six underwent VPS placement followed by CP as separate procedures (staged group).

The simultaneous group comprised four males and two females, with ages ranging from 0.5 to 8 years. The staged group had a median age of 8.5 years (range, 4–15 years).

At initial presentation, five patients in the simultaneous group had severe neurological impairment (a GCS score of 3 in four patients and 6 in one), whereas one patient had a GCS score of 15. The median initial GCS score was 3 (range, 3–15) in the simultaneous group and 3.5 (range, 3–7) in the staged group, with no statistically significant difference in this pilot sample (exact permutation Mann–Whitney test, *p* = 0.924).

In the staged group, the underlying pathology consisted of acute hemispheric subdural hematoma in five patients and traumatic subarachnoid hemorrhage in one. All cranioplasties were performed using autologous bone flaps (Table 2).

### 3.2. Timing of Surgery

In the simultaneous group, the interval between DC and combined CP–VPS surgery ranged from 26 to 229 days, with a median of 56 days. Three patients underwent combined surgery within approximately five weeks of DC, whereas surgery was delayed for several months in one patient because of clinical considerations.

In the staged group, the median interval from DC to VPS placement was 89.5 days (range, 41–160 days), and the median interval from VPS placement to subsequent CP was 298.5 days (range, 129–432 days). The overall interval from DC to CP was 414 days (range, 228–509 days).

### 3.3. Postoperative Complications

Postoperative complications occurred in two patients in the simultaneous group (2/6, 33.3%). One patient developed a wound infection requiring revision and antibiotic treatment, and another experienced shunt dysfunction requiring surgical revision. No intracranial hemorrhage or deep shunt infection was observed.

Complications were also recorded in two patients in the staged group (2/6, 33.3%), comprising one wound infection and one shunt dysfunction. The overall complication rates did not differ between the groups (Fisher’s exact test, *p* = 1.000). No intracranial hemorrhage or deep shunt infection was recorded in the staged group.

### 3.4. Neurological Outcomes

Neurological outcomes were poor overall in the simultaneous group. At the last neurological assessment, three patients had a Glasgow Outcome Scale (GOS) score of 3 and three had a GOS score of 2, corresponding to a median GOS score of 2.5 (range, 2–3). Two patients subsequently died during follow-up because of their underlying neurological conditions.

In the staged group, final GOS scores were 5, 3, 4, 1, 2, and 3, corresponding to a median of 3.0 (range, 1–5). The distribution of final GOS scores did not differ significantly between the simultaneous and staged groups (exact permutation Mann–Whitney test, *p* = 0.457).

Given the very small sample size, these analyses were exploratory and descriptive. The absence of statistically significant differences should not be interpreted as evidence of equivalence or comparable safety between the two surgical strategies.

## 4. Discussion

The optimal timing and sequencing of cranioplasty (CP) and ventriculoperitoneal shunt (VPS) placement following decompressive craniectomy (DC) remain controversial, particularly in pediatric patients. Performing both procedures during the same operation may reduce cumulative exposure to anesthesia, avoid an additional surgical admission, and potentially facilitate earlier neurological rehabilitation. Restoration of cranial integrity may also improve cerebral perfusion and cerebrospinal fluid (CSF) dynamics. Conversely, combining cranial reconstruction with permanent CSF diversion may increase procedural complexity and raise concerns regarding infection, shunt dysfunction, hemorrhage, overdrainage, and subdural fluid collections [17].

In the present pilot cohort, postoperative complications occurred in two of six patients (33.3%) in the simultaneous group and two of six patients (33.3%) in the staged group. Each group included one wound infection and one shunt dysfunction, while no intracranial hemorrhage or deep shunt infection was recorded. Although the observed complication rates were identical and Fisher’s exact test showed no statistically significant difference (*p* = 1.000), this finding should not be interpreted as evidence that the two strategies have equivalent safety. With only six patients in each group, the study had very limited ability to detect clinically meaningful differences, particularly for uncommon complications. The results therefore provide descriptive institutional experience rather than evidence supporting the superiority, non-inferiority, or equivalence of either approach [16].

The timing data illustrate an important practical difference between the two strategies. In the simultaneous group, the median interval from DC to combined CP–VPS surgery was 56 days, with a range of 26–229 days. In the staged group, the median interval from DC to VPS placement was 89.5 days, followed by a median interval of 298.5 days between VPS placement and CP. Consequently, the median overall interval from DC to cranial reconstruction in the staged group was 414 days. These observations suggest that simultaneous surgery may allow earlier restoration of cranial integrity in selected patients. However, this apparent temporal advantage cannot be interpreted causally because the choice of surgical strategy was not randomized and was likely influenced by clinical stability, infection risk, neurological condition, hydrocephalus severity, and readiness for cranioplasty. Moreover, a formal comparison of surgical intervals could not be performed using the available patient-level data [1].

Neurological outcome appeared to be influenced predominantly by the severity of the underlying brain injury. The median final Glasgow Outcome Scale (GOS) score was 2.5 in the simultaneous group and 3.0 in the staged group, without a statistically significant difference (*p* = 0.457). Two patients in the simultaneous group subsequently died because of their underlying neurological conditions. The broad distribution of outcomes in the staged group, with GOS scores ranging from 1 to 5, further reflects the clinical heterogeneity of this population. Similarly, baseline neurological status did not differ significantly between the groups (*p* = 0.924). Nevertheless, the limited sample size and differences in the underlying pathologies preclude adjustment for injury severity or other prognostic factors. Accordingly, the neurological findings should not be attributed directly to the timing or sequencing of CP and VPS placement [14].

Pediatric patients constitute a distinct population and should not be considered a smaller extension of adult cohorts. Ongoing skull growth, age-dependent changes in cranial geometry, greater brain and cranial compliance, evolving CSF physiology, and the potential for prolonged shunt dependency may influence both surgical risk and long-term recovery. The biomechanical effects of restoring cranial vault rigidity may also differ across infancy, childhood, and adolescence. When CP is combined with CSF diversion, abrupt changes in intracranial compliance and pressure gradients could theoretically contribute to overdrainage, subdural collections, or shunt-related complications. The use of programmable valves may help manage these changes by allowing postoperative adjustment of opening pressure, but it does not eliminate the need for careful clinical and radiological monitoring [10].

The etiology of the initial neurological injury also deserves consideration. In the staged cohort, five patients underwent DC for acute hemispheric subdural hematoma and one for traumatic subarachnoid hemorrhage. Differences in traumatic injury patterns, associated parenchymal damage, CSF absorptive dysfunction, infection exposure, and neurological reserve may affect both the development of hydrocephalus and subsequent recovery. Because the present cohort was small and etiologically heterogeneous, it was not possible to examine the independent effects of age, diagnosis, injury severity, or hydrocephalus mechanism. These factors may partly explain the variability in outcomes and limit direct comparison with adult studies or other pediatric series [7].

The existing literature comparing simultaneous and staged procedures is heterogeneous. Differences in patient age, hydrocephalus etiology, surgical indications, implant material, valve type, operative sequence, antibiotic protocols, and definitions of complications complicate comparisons across studies. Some reports have described increased infectious or overall complication rates following simultaneous procedures, whereas others have reported acceptable outcomes with careful patient selection and meticulous operative technique. Pediatric-specific comparative evidence remains particularly limited, and the absence of adequately powered prospective studies prevents definitive recommendations regarding the optimal strategy [9].

In clinical practice, the decision should therefore remain individualized. Simultaneous CP and VPS placement may be considered in carefully selected children who have established hydrocephalus requiring permanent CSF diversion, are suitable candidates for cranial reconstruction, and have no evidence of active systemic, cranial, or CSF infection. Strict separation of the cranial and abdominal operative fields, appropriate antimicrobial prophylaxis, careful adjustment of programmable valve pressure, and close postoperative surveillance are important components of this approach. A staged strategy may remain preferable when infection risk is elevated, the patient is clinically unstable, the need for permanent CSF diversion remains uncertain, or the condition of the scalp and autologous bone flap is unsuitable for immediate reconstruction [13].

### Limitations

This study has several important limitations. It was retrospective, conducted at a single center, and included only 12 patients. The small sample size substantially limited statistical power and prevented multivariable adjustment for potential confounders. Surgical allocation was non-randomized and therefore susceptible to selection bias and confounding by indication. The groups were heterogeneous in age, underlying pathology, neurological severity, and operative timing. In addition, the available dataset did not permit inferential comparisons of age or surgical intervals, and longer-term outcomes relevant to pediatric patients—such as skull growth, bone-flap resorption, shunt dependency, neurodevelopment, and quality of life—could not be comprehensively evaluated.

## 5. Conclusions

Simultaneous cranioplasty (CP) and ventriculoperitoneal shunt (VPS) placement after decompressive craniectomy (DC) in pediatric patients is a feasible strategy but remains associated with a considerable risk of postoperative complications. In our series, complication rates were consistent with those reported in the literature, and overall neurological outcomes were largely determined by the severity of the primary brain injury rather than the timing strategy itself. Careful patient selection, meticulous surgical technique, appropriate valve management, and close postoperative monitoring are essential when considering a single-stage approach. While simultaneous surgery may offer logistical and rehabilitative advantages in clinically stable children with confirmed hydrocephalus, a staged strategy may be preferable in higher-risk cases. Larger multicenter pediatric studies are needed to establish evidence-based guidelines and optimize outcomes in this challenging population.

## Figures and Tables

**Table 1:** Summary of previously published studies comparing simultaneous versus staged cranioplasty and ventriculoperitoneal shunt placement.

Study (Year)	Design and Patient Population	Sample Size (Simultaneous vs. Staged)	Findings
Heo et al. (2014) [4]	Retrospective, single-center, mixed etiologies No pediatric subgroup reported	32 vs. 19	Higher reoperation, subdural hematoma, and infection rates in the simultaneous group (56% vs. 21%)
Pachatouridis D. (2014) [5]	Retrospective, single-center, Greece, mixed etiologies No pediatric subgroup reported	11 vs. 12	Higher reoperation rate in the simultaneous group
Schuss et al. (2015) [6]	Retrospective, single-center, Germany, mixed etiologies No pediatric subgroup reported	17 vs. 24	Simultaneous CP and VPS placement was associated with a significantly higher risk of postoperative complications than staged surgery
Jung et al. (2015) [7]	Retrospective, single-center, Korea, small sample No pediatric subgroup reported	~9 vs. 10	Higher complications in the simultaneous group
Von der Brelie et al. (2016) [8]	Retrospective, single-center, Germany, TBI-focused No pediatric subgroup reported	10 vs. 27	Simultaneous VP shunt placement during cranioplasty was associated with a significantly higher rate of relevant complications in the univariate analysis (*p* = 0.01)
Meyer et al. (2017) [9]	Retrospective, single-center, USA, mixed etiologies No pediatric subgroup reported	26 vs. 24	No significant difference in the complication rate between the simultaneous and staged groups (15% vs. 29%, respectively; *p* = 0.24)
Yang et al. (2017) [10]	Retrospective, single-center, China, mixed etiologies No pediatric subgroup reported	20 vs. 38	The two groups did not significantly differ regarding the all-complication (30% vs. 13%); however, the rate of surgical site infection/incision healing problems (25% vs. 3%) and the re-operation rate (20% vs. 3%) were significantly higher in the simultaneous operation group
Rosinski et al. (2020) [11]	Retrospective, single-center, USA, mixed etiologies No pediatric subgroup reported	18 vs. 22	The rates of infection, resorption, and reoperation did not differ significantly, although reoperation showed a trend toward occurring less frequently in the simultaneous group
Jung et al. (2020) (Meta) [12]	Meta-analysis of 7 studies, mixed etiologies No pediatric-specific analysis	135 vs. 256	Higher infection rates in the simultaneous group (6.2% vs. 23.7%; OR ~2.7, *p* < 0.01)
Ting CW et al. (2020) [13]	Retrospective, single-center, mixed etiologies No pediatric-specific analysis	17 vs. 32	No significant difference in surgical complications (*p* = 0.11)
Gill et al. (2021) [14]	Retrospective, single-center, Korea, mixed etiologies No pediatric-specific analysis	18 vs. 63	Higher overall complication rates in the simultaneous group (33.3% vs. 9.6%; *p* < 0.01)
Zhang et al. (2020) [15]	Retrospective, single-center, China, mixed etiologies No pediatric-specific analysis	22 vs. 64	No statistically significant difference was observed in overall complication rates; the infection rate was significantly higher in the simultaneous group (22.7% vs. 4.7%; *p* = 0.024)
Wang et al. (2023) [16]	Retrospective, single-center, China, trauma cases No pediatric-specific analysis	50 vs. 20	Improved simultaneous CP and VPS was safe, with complication rates comparable to staged surgery (22% vs. 25%; *p* = 0.763)
Zhou et al. (2022) (Meta) [3]	Meta-analysis of 12 studies, mixed etiologies No pediatric-specific analysis	236 vs. 415	Higher overall complication rates in the simultaneous group (pooled OR = 2.0; *p* < 0.05)
Zhang et al. (2023) (Meta) [2]	Meta-analysis of 10 studies, mixed etiologies No pediatric-specific analysis	193 vs. 322	Staged surgery showed less postoperative subdural effusion, although this finding was not robust; other complication rates did not differ significantly

Abbreviations: CP, cranioplasty; VPS, ventriculoperitoneal shunt; TBI, traumatic brain injury; ICH, intracranial hemorrhage; OR, odds ratio.

**Table 2:** Summary of patient characteristics of the simultaneous group (n = 6). (GCS: Glasgow Coma Scale, GOS, Glasgow Outcome Scale). * Patients 1 and 2 died during follow-up; however, their last recorded GOS before death was 2 and 3, respectively; these outcome scores are distinct from the last recorded GCS values presented in the table.

Patient	Age (yrs)	Sex	Interval from DC to Surgery (Days)	Postoperative Complications	GCS at Initial and Last Follow-Up	Follow Up Duration (Month)	Indication
1 *	0.5	F	50	None	3–3	3	Acute subdural hematoma in the right hemisphere + acute subdural hematoma and epidural hematoma in the left hemisphere
2 *	3	M	73	None	3–5	5	Bifrontal Contusion + Widespread Traumatic SAH
3	5	M	26	Wound infection	3–12	72	Left hemispheric acute subdural hematoma, extensive traumatic subarachnoid hemorrhage and contusion
4	2	M	35	None	15–15	61	Acute left hemispheric subdural hematoma
5	8	M	229	Shunt dysfunction	6–14	78	Right hemispheric infarction + traumatic SAH
6	2	F	63	None	3–13	80	Widespread Left Traumatic SAH

## Data Availability

The original contributions presented in this study are included in the article. Further inquiries can be directed to the corresponding author.
